# A possible role for *Phlebotomus *(*Anaphlebotomus*) *rodhaini *(Parrot, 1930) in transmission of *Leishmania donovani*

**DOI:** 10.1186/1756-3305-4-238

**Published:** 2011-12-21

**Authors:** Dia-Eldin A Elnaiem, Hassan K Hassan, Omran F Osman, Rhayza DC Maingon, Robert Killick-Kendrick, Richard D Ward

**Affiliations:** 1 Department of Natural Sciences, University of Maryland Eastern Shore, 1 Backbone Rd, Princess Anne, MD 21853, USA; 2 Department of Zoology, University of Khartoum, Khartoum P O Box 321, Sudan; 3 College of Public Health, University of South Florida USA; 4 School of Life Sciences, Keele University, Staffordshire ST5 5BG, UK; 5 Imperial College at Silwood Park, Ascot, UK

**Keywords:** *Leishmania donovani*, transmission, sand flies, *Phlebotomus rodhaini*, visceral leishmaniasis

## Abstract

**Background:**

Visceral leishmaniasis (VL, kala azar), caused by *Leishmania donovani *is a major health problem in Sudan and other East African countries. In this region the only proven vectors of *L. donovani *are *Phlebotomus orientalis *in eastern Sudan, Ethiopia and Upper Nile areas of Southern Sudan and *Phlebotomus martini *in Ethiopia, Kenya and Southern Sudan. In this report, we present the first evidence that *Phlebotomus rodhaini *may also play a role in maintaining transmission of *L. donovani *between animal reservoir hosts in eastern Sudan. The study was conducted in a zoonotic focus of visceral leishmaniasis in Dinder National Park, eastern Sudan, where previous work showed high infection rates of *L. donovani *in *P. orientalis*. Sand flies, captured by CDC traps were dissected and examined for infection with *Leishmania *parasites. Parasite isolates were subjected to *L. donovani *specific PCR. Field experiments were also carried out to compare efficiency of rodent baited and un-baited CDC traps in collection of *P. rodhaini *and determine its man-biting rate.

**Results:**

Three female *P. rodhain*i were found infected with *Leishmania *parasites in an astonishingly small number of flies captured in three separate field trips. Two of these isolates were typed by molecular methods as *L. donovani*, while the third isolate was inoculated into a hamster that was subsequently lost. Although *P. rodhaini is *generally considered a rare species, results obtained in this study indicate that it can readily be captured by rodent-baited traps. Results of human landing collection showed that it rarely bites humans in the area.

**Conclusion:**

It is concluded that *P. rodhaini *is a possible vector of *L. donovani *between animal reservoir hosts but is not responsible for infecting humans. It is suggested that the role of *P*. *rodhaini *in transmission of *L. donovani *in other zoonotic foci of visceral leishmaniasis in Africa should be re-examined.

## Background

The epidemiology of vector-borne diseases, such as leishmaniasis, is governed by the presence of specific vector species that are capable of supporting the development of a pathogen and transmitting it to a susceptible host. Therefore, proper knowledge of natural vectors is a fundamental pre-requisite for understanding the transmission dynamics of these diseases and planning adequate control programs. Although the main transmission cycles have been described for most vector-borne diseases, there is a possibility of existence of "hidden" vectors that are missed due to employment of an inadequate range of trapping methods.

Leishmaniasis is a group of diseases caused by protozoan parasites of the genus *Leishmania *(order: Kinetoplastida; family: Trypanosomatidae) that are transmitted between humans and other mammalian hosts by phlebotomine sand flies (Order: Diptera; family: Psychodidae). Depending on the species of *Leishmania *parasites and other immunological and epidemiological factors, *Leishmania *infection can lead to cutaneous (CL), mucocutaneous (MCL) or visceral leishmaniasis (VL, kala azar).

Visceral leishmaniasis is a deadly form of leishmaniasis that affects approximately 500,000 people each year [1-5]. Caused by members of *L. donovani *complex that are transmitted by several species of sand flies, VL is a systemic disease characterized by a range of symptoms including fever, hepatospleenomegaly, lymphadenopathy, weight loss, weakness and death, if left untreated [3]. The transmission cycles of parasites causing visceral leishmaniasis may be strictly zoonotic as for *L. infantum *in Latin America, southern Europe and the Mediterranean region or strictly anthroponotic as for *L. donovani *in the Indian subcontinent or may include both anthroponosis and zoonosis as for *L. donovani *in East Africa [6].

Despite the wide spread distribution of visceral leishmaniasis, relatively few species of sand flies transmit *Leishmania donovani *parasites in nature, and there is a parallel evolution of sand fly species and the *Leishmania *parasites they transmit [7,8]. In their vectors, *Leishmania *parasites undergo an obligatory multiplication and maturation cycle that culminates in the production of an infective metacyclic promastigote stage [9-11]. For members of the *L. donovani *complex, most Old World sand fly species that satisfy these criteria fall within the subgenus Larroussius that includes most vectors of *L. infantum *in the Mediterranean region, and *P. orientalis*, the known vector of *L. donovani *in East Africa. Other subgenera of Old World sand flies that include vectors of *L. donovani *are Euophlebotomus that contain *P. argentipes *in India and Synphlebotomus that include *P. martini*, and *P. celiae *in East Africa.

Some of the most important foci of visceral leishmaniasis occur in East Africa, where the disease has recently claimed the lives of hundreds of thousands of people in Sudan [12,13]. In this region, the transmission of *L. donovani *is thought to occur through an anthroponotic cycle [14]. However, there is strong evidence for existence of zoonotic cycles both in the *Acacia seyal/Balanites aegyptiaca *woodland and the village habitats of eastern Sudan. In a previous study we encountered high infection rates of *L. donovani *in *P. orientalis *sand flies in uninhabited woodland in Dinder National Park (DNP), eastern Sudan, indicating a close association between this vector and a sylvatic reservoir host [15]. In a subsequent study, we found two out of 13 individuals of the Egyptian mongoose (*Herpestes ichneumon*) infected with *L. donovani*, indicating that this animal may be a reservoir host for the parasite [16]. In other studies in villages lying about 100 km away from our study area, Dereure *et al*. [17] showed high prevalence of *L. donovani *infection in dogs.

The only proven vectors of *L. donovani *in East Africa are *P. orientalis *in Eastern Sudan, Upper Nile region of Southern Sudan and parts of Ethiopia and *P. martini *in Ethiopia, Kenya and Sudan/Kenya border area in southern Sudan [6,14-16,18-21]. In this paper we present the first evidence that *P. rodhaini *may be involved in maintaining the transmission of *L. donovani *between wild animals and dogs. We also investigate the man-biting rates of *P. rodhaini *and the effects of rodent-baited traps in sampling this sand fly species.

## Results

*Leishmania *promastigotes were found in 3 females of *P. rodhaini *collected in 3 separate field trips in a fixed site in DNP (Table 1). Mature heavy infection of the parasite was observed in the three specimens that were carefully examined for parasite form, location and density. Large numbers of highly motile metacyclic promastigotes were located in the anterior part of the thoracic midgut and also throughout the alimentary tract. The infection loads were similar to those seen in *P. orientalis*, the proven vector of *L. donovani *in the area [14-16]. Unfortunately, the hamster used for inoculation of promastigotes from our first infected sand fly died after three months, resulting in the loss of the parasite isolate. However, the PCR performed on the two isolates made in 1998 and 1999 resulted in bands specific for *L. donovani*. Further confirmation of the identity of the parasite was obtained following Southern blotting and hybridization of PCR products with an *L. donovani *specific probe.

**Table 1:** Records of infections of *Leishmania donovani *in *Phlebotomus rodhaini *collected from DNP*, eastern Sudan

Date of collection	Method of collection	Total No. of *P. rodhaini *examined	No. of infected *P. rodhaini *females	*L. donovani *specific PCR confirmation
March 1996	CDC light traps	15	1	Not done

March 1998	CDC light traps	2	1	Positive

May 1999	CDC light traps	3	1	Positive

Total	CDC light traps	20	3	--

*DNP = Dinder National Park

The numbers of *P. rodhaini *collected and examined for infection were too small to allow estimation of infection rates. However, it was remarkable that the infection was repeatedly seen in an extremely small number of flies, indicating a high infection rate.

The rodent-baited trap experiment conducted in this study clearly showed that *P. rodhaini *is captured in larger numbers when traps were baited with an animal (Table 2), increasing from an average of 0.25 flies in un-baited CDC traps to 10 females per rodent baited trap. These trap yields contrasted sharply with those of *P. orientalis*, which was captured in high numbers in CDC traps and showed relatively little attraction to the rodent-baited trap.

**Table 2:** Numbers of *Phlebotomus *sand flies caught in rodent-baited and un-baited traps in DNP, eastern Sudan.

Trap Type	**Experiment Replicate No**.	*Phlebotomus rodhaini*			*Phlebotomus orientalis*		
		
		male	female	total	male	female	total
Rodent baited CDC trap without Light	1	2	6	8	1	0	1
	
	2	3	8	11	1	3	4
	
	3	2	4	6	10	5	15
	
	4	3	12	15	0	2	2
	
	Total	10	30	40	12	10	22
	
	Average	2.5	7.5	10	3	2.5	5.5

Control CDC trap Without rodent and without light	1	0	0	0	0	1	1
	
	2	0	1	1	1	2	3
	
	3	0	0	0	1	0	1
	
	4	0	0	0	0	1	1
	
	Total	0	1	1	2	4	6
	
	Average	0	0.25	0.25	0.5	1	1.5

Control CDC trap Without rodent and with light	1	0	0	0	30	22	52
	
	2	0	0	0	21	32	53
	
	3	0	1	1	39	14	53
	
	4	0	0	0	30	11	41
	
	Total	0	1	1	120	79	199
	
	Average	0	0.25	0.25	30	19.25	49.25

*DNP = Dinder National Park

*Phlebotomus rodhaini *was rarely encountered in human landing collections during two field investigations on man biting sand flies in DNP. In the first trip, no *P. rodhaini *was captured during 6 night 2-h trapping events each involving 4 volunteers. In this collection the only sand fly species encountered in the human-landing collection was *Phlebotomus orientalis*, which exhibited a mean human landing rate of 5.7 females per man/2h/night. Since trapping was restricted to 2 h in the early evening, we were concerned that *P. rodhaini *may bite humans late at night. However, when whole night human landing collections were done in the subsequent field trip, only two female *P. rodhaini *were caught by 3 volunteers in six trapping nights, resulting in 0.1 female *P. rodhaini */ man/night; as compared to 32 female *P. orientalis */ man/night.

## Discussion

The results obtained in this study indicate that *Phlebotomus rodhaini *may be a vector of *L*. *donovani *in eastern Sudan and possibly other areas in Africa. The specific PCR assay conducted on two of the 3 isolates confirmed the identity of the parasite found in *P. rodhaini *as *L. donovani*. Although the loss of the parasite isolate from the first fly precluded subsequent biochemical and molecular characterization, we have two strong reasons to suggest that this isolate was also *L. donovani*. Firstly the morphology of the isolated promastigotes and their location in the anterior part of the alimentary tract strongly indicated that the parasite was of the genus *Leishmania*. Secondly, *L. donovani is *the only *Leishmania *parasite found in the study site and other areas in eastern Sudan and it was previously isolated from a large number of *P*. *orientalis *in the same collection [16].

This is the first record of natural infection of *Leishmania *parasites in *P. rodhaini *and any *Anaphlebotomus *sand flies. Despite its wide distribution in most leishmaniasis endemic foci, *P. rodhaini *is considered a rare species and therefore it was ignored as a probable vector of *Leishmania *parasites. However, from the results of the rodent-baited traps shown here, it seems that the abundance of *P. rodhani *was previously underestimated because of inadequate collection techniques. It is noteworthy that from a whole year collection using large numbers of sticky paper traps and CDC light traps at indoor and outdoor sites of a village and also in DNP, we have previously obtained a total of 18 individuals of *P. rodhaini *[22]. However, the use of rodent baited traps in this study increased the collection of *P. rodhaini *by more than 40 fold. This result supports the findings of Hoogstraal & Heyneman [18], who reported that *P. rodhaini *is more readily collected by rodent baited traps. Although in the current study, the collection of *P. rodhaini *in rodent-baited traps still remained at a relatively low average number of 10 per trap night, the results show clearly that CDC light traps are not suitable for collection of this sand fly species. Interestingly, in a recent study we have found that even higher numbers of *P. rodhaini *were captured when traps were baited with dogs (*Canis familiaris) *or the Egyptian mongoose (*Herpestes ichneumon*) instead of the Nile rat (*Arvicanthis niloticus*) [23]. In this study, the collection of *P. rodhaini *increased from an average of 0.1 fly per CDC light trap without a light bulb to 1.0 per CDC trap baited with a Nile rat baited trap to 3.2 in a trap-baited with a genet to 5.88 to trap baited with a mongoose and to 29.7 in a dog baited trap. These astonishing results indicate that *P. rodhaini *is attracted in even higher numbers to traps baited by probable reservoirs of *L. donovani*

Our data on human landing collections showed that *P. rodhaini *has a low man-biting rate in the area. These observations indicate that the role of *P. rodhaini *in the epidemiology of visceral leishmaniasis in the area may be limited to transmission of *L. donovani *between reservoir animals, with rare infection taking place on people. This contrasts with the role of *P. orientalis*, which is responsible for transmission both between animals and from animals to humans that intrude in the cycle.

The results of the current study should have important implications in understanding the epidemiology of leishmaniasis in other places of Africa. *Phlebotomus rodhaini *has a wide distribution in the continent and it is encountered in sporadic foci of canine leishmaniasis in West Africa, where no known vectors of *L. donovani *are found. Recently, Senghor *et al*. used sticky paper traps, CDC traps and indoor knock down collection to capture sand flies in 16 villages with high endemicity of canine leishmaniasis in Senegal [24]. Based on its rare presence in their collection, *P. rodhaini *was ruled out as a possible vector of the parasite. Alternatively, the authors hypothesized that the vector is likely to be one or more of the highly abundant *Sergentomyia *sp; mainly *S. dubia *and *S. schwetzi*, which were encountered in large numbers in areas with high seroprevalence of *L. donovani *in dogs. Although the hypothesis of these authors may still be valid, we strongly suggest use of dog baited traps for collection of sand flies, before ruling out the possibility that *P. rodhaini *is a probable vector of *L. donovani *in these foci. As shown above, none of the classical sand fly collection methods are suitable for sampling of *P. rodhaini *and conclusions on its abundance in different foci of Africa should be re-examined.

Incrimination of vectors of *Leishmania *parasites involves a number of criteria that include abundance in a leishmaniasis focus, high human biting rates, demonstration of natural infections and the ability to transmit the parasite to a susceptible host [7,8]. The natural heavy mature infection of *L. donovani *observed in this study indicates that *P. rodhaini *has the ability to transmit the parasite to a susceptible host. However, future laboratory work will be needed to study the development of *L. donovani *in *P. rodhaini *and determine the efficiency of its transmission by this potential new vector. Further work is also urgently needed to determine the infection rates of *L. donovani *in *P. rodhaini *in the study area and other endemic foci and incriminate the reservoir hosts of the parasite.

## Conclusions

In conclusion, this study provides strong evidence that *P. rodhaini *plays a role in transmission of *L. donovani *between animal reservoir hosts. Although man may accidently be bitten by *P. rodhaini*, it is unlikely that this sand fly species plays a significant role in transmitting the parasite to humans. It is suggested that further work is needed to study the parasite cycle in this new vector and determine the role of *P. rodhaini *in maintaining the transmission cycle of *L. donovani *in other places of Africa, including the West African foci of canine visceral leishmaniasis.

## Methods

### Study area

The fieldwork of this study was carried out in a woodland site in Dinder National Park (DNP) in eastern Sudan, which is an endemic focus of visceral leishmaniasis [13,15,16,25-27]. The location and ecology of the study site were described by Elnaiem *et al*. [16,22]. The land is flat, covered by black cotton soil. The climate is typical of tropical savannah woodland with an annual rainfall of 600 mm. There are three distinct climate seasons: a hot dry summer (March-May), a warm rainy season (autumn: June-October) and a moderately warm winter (November-February). The average temperature and relative humidity of the area are 32.3°C and 25.9%RH in summer, 26.2°C and 54.4%RH in autumn and 27.2°C and 29.8%RH in winter. The area lies within the DNP which is an extensive belt (3,500 km surface area) of uninhabited woodland, apart from a few game warden camps scattered at different locations. Approximately 25 wardens living in 10 huts constructed of wood and bamboo and hatched with grass temporarily inhabited the biggest of these camps, named Gelegu camp, which was located a few hundreds meters from our sand fly trapping site. Over many years, occasional cases of VL have been recorded among the game wardens providing evidence for active zoonotic transmission of *L*. *donovani *around the camp. This is supported by a relatively high rate of infection of *L. donovani *in *P. orientalis *collected from a thicket lying a few hundred meters away from the camp [15,16].

### Isolation and typing of L. donovani infections in sand flies

Infection of *L. donovani *in sand flies was surveyed during 3 field trips in March 1996, March 1998 and May 1999. In each of these surveys, sand flies were collected from the same study site by CDC light traps operated between Sunset to dawn. Captured female sand flies were dissected and examined for infection by *Leishmania *parasites as described by Elnaiem *et al*. [16]. *Leishmania*-infected sand fly midguts were carefully examined under a phase contrast microscope to determine location and density of different forms of the parasite. Parasites isolated in the first field trip were inoculated intraperitoneally into the abdomen of a three week old hamster. In the two subsequent field trips the isolated parasites were left to dry out on the slides, which were then wrapped in aluminum foil and stored at room temperature for later PCR analysis. In the laboratory, DNA from these specimens was extracted using methods described by Elnaiem & Osman [14] and Elnaiem *et al*. [16] and subjected to nested-PCR protocol developed by Noyes *et al*. [28]. Briefly, products from an initial round of PCR, using the external primers CSB2XF (C/GA/GTA/GCAGAAAC/TCCCGTTCA) and CSB1XR (ATTTTTCG/ CGA/ TTTT/ CGCAGAACG) were diluted (1:10) in molecular grade water and subjected to a second PCR reaction using the oligonucleotides 13Z (ACTGGGGGTTGGTGTAAAATAG) and LiR (TCGCAGAACGCCCCT) as internal primers. The final PCR products were subjected to electrophoresis on 1.5-% agarose gel, ethidium bromide stained and then visualized by ultraviolet illumination. *Leishmania *positive samples, showing a typical band size of 752 ± 25 bp were re-tested by *Leishmania donovani *specific kDNA-PCR primers (AJS3/ DBY: 5' GGG GTT GGT GTA AAA TAG GGC CCG 3' and DBY 5' CCA GTT TCC CGC CCC GGA G 3') described by Smyth *et al*. [29]. PCR products were subjected to electrophoresis as described, and bands of approximately 805 bp were considered specific for *L. donovani*. Controls for all PCR tests consisted of DNA from *L. donovani*, molecular grade water and DNA from *L. major*. Additionally, DNA of *L. donovani *isolated from *P. orientalis *and two midguts of *Sergentomyia schwetzi *from the study area that were found naturally infected with kinetplastid promastigotes were also included in the PCR tests. For additional confirmation of the identification of the parasites isolated from *P. rodhaini*, PCR products were subjected to Southern blotting and subsequent hybridization with an *L. donovani *specific probe as described previously [27].

### Comparison of efficiency of rodent-baited and un-baited CDC light traps in sampling P.rodhaini and P. orientalis

Attraction of *Phlebotomus *sand flies to rodent-baited CDC traps was investigated in a field experiment in DNP in March 1996. An inverted CDC light trap, from which the light bulb was removed, was operated overnight (from 18:00-06:00 HR) at 5 cm above a small cage (approx 12 × 6 × 6 cm), containing an *Arvicanthis niloticus *male captured from the same study area. As controls, two traps, one with, and the other without, a light bulb were run simultaneously. Traps were located at an approximate distance of 15 m from each other. Space between traps was covered by mature trees of *Acacia seyal *and dry grass. The experiment was replicated 4 times in 4 consecutive nights. Although traps were located within the same vicinity, effects of bias, resulting from sampling locations, was avoided by rotating the traps at each new replicate. *Phlebotomus *sand flies captured in each trap were identified to the species level, using keys of Kirk and Lewis [30] Quate [31] and Abonnenc [32]. Numbers of sand flies caught each night were tabulated according to species, sex and trap type.

### Investigations on the man-biting rates of Phlebotomus rodhaini

Man biting rates of sand flies in the area were investigated in two field trips conducted in March 1995 and June 1996. In the first trip, human landing collection was conducted by 4 volunteers for two hours, between 19:00-21:00 HR each night for six consecutive nights. In the second trip, whole night collection was carried out by 3 volunteers, between 18:00-06:00 HR, for 6 consecutive nights. In each trip, volunteers used aspirators and torch to capture sand flies alighting to bite them. Care was taken that sand flies were not allowed to bite.

### Ethical approval for studies involving animals and human

The experiments involving human volunteers and animals, described in this report, were ethically approved by the Research Board of the Faculty of Science, University of Khartoum (Sudan).

## Competing interests

The authors declare that they have no competing interests.

## Authors' contributions

DAE contributed in designing the study, conducting field and laboratory work, analysis of data and drafting the manuscript. HKH helped in the field trips, the isolation of the parasite and editing the manuscript. OFO contributed to the fieldwork and the molecular work. RDCM contributed to the molecular work done at her laboratory at Keele University (UK) and editing the manuscript. RK participated in the design of the rodent-baited traps and the development of the ideas and concepts of the study. RDW contributed to the fieldwork, the development of the ideas of the study, the interpretation of results and editing the manuscript. All authors read and approved the final version of the MS.
